# IgA Vasculitis Complicated by Both CMV Reactivation and Tuberculosis

**DOI:** 10.3390/pediatric13030048

**Published:** 2021-07-22

**Authors:** Małgorzata Mizerska-Wasiak, Maria Winiarska, Karolina Nogal, Karolina Cichoń-Kawa, Małgorzata Pańczyk-Tomaszewska, Jadwiga Małdyk

**Affiliations:** 1 Department of Pediatrics and Nephrology, Medical University of Warsaw, 02-091 Warsaw, Poland; karolina.cichon-kawa@wum.edu.pl (K.C.-K.); mpanczyk1@wum.edu.pl (M.P.-T.); 2 Student’s Scientific Group at the Department of Pediatrics and Nephrology, Medical University of Warsaw, 02-091 Warsaw, Poland; winiarska.marysia@gmail.com (M.W.); karolinanogal95@gmail.com (K.N.); 3 Department of Pathology, Medical University of Warsaw, 02-091 Warsaw, Poland; jadwiga.maldyk@wum.edu.pl

**Keywords:** tuberculosis, CMV, IgA nephropathy, IgA vasculitis, proteinuria, immunosuppressive therapy, renal function, nephrotic syndrome

## Abstract

Immunoglobulin A (IgA) vasculitis is the most common systemic vasculitis in the pediatric population. We present the case of a patient with IgA vasculitis with nephritis who developed cytomegalovirus (CMV) infection followed by Mycobacterium tuberculosis infection. In the literature, there are a few cases of IgA nephropathy accompanied by reactivation of CMV or tuberculosis. To the best of our knowledge, this is the first reported case of IgA vasculitis complicated by both CMV reactivation and tuberculosis. It is important to detect infections in patients with IgA vasculitis because they can induce and exacerbate the symptoms of the disease. Effective antimicrobial treatment facilitates the management of proteinuria and slows down the decline of renal function. Immunosuppressive therapy is a risk factor for reactivation of latent infections and makes patients more susceptible to its generalized and complicated course. This can be prevented by actively screening for hidden sites of infection.

## 1. Background

IgA vasculitis is the most common systemic vasculitis in the pediatric population. Deposition of IgA antibodies in a vascular wall causes a typical tetrad of clinical symptoms: skin lesions (purpura), joint involvement, abdominal pain, and renal symptoms (hematuria, proteinuria). Renal manifestations are most likely to occur in the first six months after the onset of IgA vasculitis, although, sometimes, they can manifest even after two to five years [1,2]. The etiology of IgA vasculitis is unknown; however, the presence of poorly galactosylated IgA1 is postulated to be one of the underlying pathophysiological factors [3]. Infections can trigger the deposition of antibodies in the vascular wall [4]. We report a case of a patient with IgA vasculitis complicated by cytomegalovirus infection and tuberculosis. A few cases of IgA nephropathy accompanied by either CMV or reactivation of tuberculosis have been reported so far [5,6,7]. To the best of our knowledge, this is the first reported case of IgA vasculitis complicated by both CMV reactivation and tuberculosis. The aim of this case is to show that screening for latent infections and early introduction of treatment are crucial for successful management of IgA vasculitis patients. The importance of it cannot be stressed enough since even subclinical infections can exacerbate the clinical course of the disease and may lead to an unsatisfactory response to immunosuppressive treatment.

## 2. Case Presentation

A fourteen-year-old patient presented to the hospital with a seven-day history of abdominal pain. He also reported blood-stained vomiting once or twice a day. He was diagnosed with appendicitis and underwent an appendectomy. The day after the surgery, he developed symmetrical purpura on legs and buttocks, and the characteristics of the lesion raised the suspicion of IgA vasculitis.

The following day, he complained of severe abdominal pain, and paracetamol was administered. He was given hydrocortisone 4 × 150 mg, and his general condition improved. On the fourth postoperative day, joint swelling of elbow, wrist, ankle, and thumb occurred. His renal function deteriorated; the creatinine level was 0.88 mg/dL (GFR 82 mL/min/1.73 m^2^), proteinuria of 59.3 mg/dL, hematuria of 25–30 HPF. The patient was transferred to the Nephrology Department, where further workup confirmed the diagnosis of IgA vasculitis.

On admission, the test results revealed increased serum levels of IgA, creatinine 0.8 mg/dL (GFR 90 mL/min/1.73 m^2^), proteinuria 24 mg/kg/day, and albumin 1.8 g/dL. The cholesterol level was not elevated. Complement and antinuclear antibody (ANA) levels were normal. The values are shown in Table 1.

Despite the treatment with the highest doses of prednisone (2 mg/kg/day), the symptoms increased, and nephrotic syndrome was observed (proteinuria > 50 mg/kg/day, hypoalbuminemia < 2.5 g/dL, hyperlipidemia, and oedema) a few days later. Generalized edema and hydrothorax were present, and the test results showed proteinuria (459 mg/kg/day) and hypercholesterolemia (496 mg/dL), and the albumin level was low (1.8 g/dL). Methylprednisolone pulses (17 mg/kg/dose) were administered, but the treatment was discontinued after two doses because of low-grade fever. Kidney biopsy was performed in the second week of nephrotic syndrome. A total of six glomeruli were identified, all showing mesangial expansion with hypercellularity and endocapillary proliferation. Focal necrosis and interstitial fibrosis were also observed. One of the glomeruli showed segmental sclerosis. Immunofluorescence microscopy showed IgA + 4 granular deposits in the glomerular mesangium. Diagnosis: IgA nephropathy, according to the Oxford classification M1E1S1T1C0.

**Figure 1:**
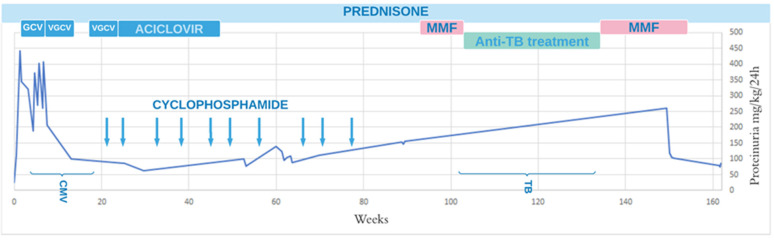
Treatment and proteinuria (mg/kg/day). GCV, ganciclovir; VGCV, valganciclovir; CMV, Cytomegalovirus; TB, tuberculosis.

Due to nephrotic syndrome, the treatment was changed to azathioprine 2 mg/kg/day combined with prednisone 60 mg/day. Since the patient had low-grade fever, he was tested for possible infections. The results confirmed active CMV infection. The patient was treated with ganciclovir, and both his general condition and kidney function improved, while proteinuria decreased to 244 mg/kg/day. After 3 weeks of treatment, a viral load of 6000 copies was observed, and valganciclovir 900 mg/day was administered for the following 56 days. Five months after the first admission to the hospital, no viral DNA was found. Prophylactically, Aciclovir at a dose of 800 mg/day was given, and after a month, it was reduced to 600 mg/day, which was continued for the next 2 years. At this period, proteinuria levels were still high, 5 g/day (100 mg/kg/day), and therefore, azathioprine was changed to pulses of cyclophosphamide (155 mg/kg/treatment). In total, 10 pulses were administered monthly, the last one 20 weeks after the diagnosis. After 10 cycles of cyclophosphamide pulses, proteinuria levels reached 9 g/day (150 mg/kg/day). Therefore, mycophenolate mofetil (MMF) was introduced for 2 weeks combined with 25 mg/48 h prednisone. The patient was tested for the presence of an infection, and a positive result of the Quantiferon test was obtained. Chest X-ray was normal. Treatment with MMF was discontinued, and the patient was referred to the Tuberculosis Treatment Center, where the infection was confirmed. Prior to the diagnosis, the patient was not exposed to anyone with active tuberculosis. Isoniazid (INH) 300 mg, rifampicin (RMP) 600 mg, and pyrazinamide (PZA) 1500 mg were administered during a 2-month intensive phase, followed by a 4-month continuation phase with INH and RMP with the same doses. When antituberculosis (TB) treatment was completed, the therapy with 20 mg/48 h prednisone, and 1500 mg MMF (1000 mg/m^2^/day) was continued; proteinuria was reduced to 70 mg/kg/day, and kidney function was satisfactory. The treatment and its influence on proteinuria are shown in Figure 1.

## 3. Discussion

The presented case shows the first report of the coexistence of IgA vasculitis with CMV infection and subsequent Mycobacterium tuberculosis infection. The etiology of IgA vasculitis is unknown. The pathogenesis is most likely multifactorial, and genetic predisposition may contribute to its development [3]. Infections can induce and aggravate the symptoms of IgA vasculitis [4]. Symptoms may resolve spontaneously; however, some patients develop hematuria, as well as proteinuria, and renal biopsy reveals changes similar to those typically observed in IgA nephropathy [8]. Initial presentation of IgA vasculitis with nephrotic syndrome, elevated serum creatinine, and extensive changes in renal biopsy are associated with unfavorable kidney outcomes, including end-stage renal failure [9]. Our patient presented with nephrotic syndrome (proteinuria > 50 mg/kg/day, hypoalbuminemia < 2.5 g/dL, hyperlipidemia, and oedema) and high serum creatinine levels. Levels of proteinuria and serum creatinine tended to normalize when an effective treatment for coexisting latent infection was initiated. Our case presents the importance of identifying hidden sites of infection, considering that even mildly symptomatic infections can aggravate the course of underlying disease and contribute to the deterioration of kidney function.

The patient first presented with symptoms of appendicitis and underwent an appendectomy. This raises the question whether the appendicitis triggered a stress response leading to activation of IgA vasculitis or if it was its first manifestation. At the moment of surgery, the patient did not show symptoms raising suspicion of IgA vasculitis. The patient underwent surgery at another hospital where appendicitis simplex was diagnosed. The inflammatory process in the appendix may have caused autoimmune vasculitis.

However, there are known cases where IgA vasculitis first manifested as appendicitis. In that case, IgA immune complexes deposit in the appendix, which is not typical for acute appendicitis [10]. Appendicitis itself can also be a very rare complication of the underlying vascular disease [11].

Approximately ⅓ of the world’s population is infected with M. tuberculosis (MTB). Although there are effective antituberculosis drugs, timely TB detection and treatment can pose a diagnostic challenge. The treatment duration is long and, in pulmonary tuberculosis, typically six months. It consists of four or three drugs in the induction phase (isoniazid + rifampicin + pyrazinamide +/− ethambutol), and after two months, it is limited to two drugs (isoniazid + rifampicin) [12]. MTB associated with IgA nephropathy has been reported in the literature. An active tuberculosis is associated with increased serum IgA levels due to the production of specific IgA against mycobacterial A60 antigen. These circulating immune complexes with IgA and mycobacterial antigens deposit in the kidney, which may aggravate symptoms of IgA nephropathy. Therefore, treatment with antituberculosis medication will help to improve impaired kidney function. An important role of the humoral response in MTB infection has also been postulated recently [13].

CMV infections are still a common problem in patients undergoing immunosuppressive therapy. The treatment is long term. The example of our patient shows how important it is to regularly control the viral load and adjust the treatment based on the viral load and the patient’s general condition. The treatment of CMV infection in immunosuppressed patients should start with ganciclovir (5 mg/kg/day intravenously) as an induction phase. Valganciclovir is used for maintenance therapy [14]. In our case, the implemented antiviral and immunosuppressive treatment successively improved the patient’s clinical condition and laboratory findings. A careful approach and treatment are vital, as it was shown that CMV reactivation in the course of IgA vasculitis may result in fatal intracranial bleeding [5].

## 4. Conclusions

Our case demonstrates that ruling out infections and treating them are very important in managing a patient with IgA vasculitis nephritis, because they can improve both the control and outcome of the vascular disease. The treatment of nephropathy associated with IgA vasculitis includes immunosuppression, which may predispose to reactivation of latent infections. Additionally, infections are a common and substantial complication of nephrotic syndrome. Our patient’s underlying disease and applied treatment made him especially vulnerable to developing such complications. Special attention should be paid to the early detection of infections. Their presence should always be taken into consideration in cases when, despite properly applied treatment, the renal function improvement is unsatisfactory and with worsening proteinuria. It is important to keep in mind that symptoms may not be specific, and therefore, the diagnosis may be difficult. Delay in diagnosis, especially in the course of active immunosuppressive treatment, can result in worsening of the course of infections and underlying disease.

## Figures and Tables

**Table 1:** Laboratory workup on admission.

Parameter	Patient’s Results	Normal Values
WBC (thou/µL)	10.3	4.0–10.0
Hb (g/dL)	10.4	14.0–18.0
PLT (thou/µL)	325	140–400
CRP (mg/dL)	1.3	0.0–1.0
albumin (g/dL)	2.4	3.7–5.6
total protein (g/dL)	4.5	6.0–8.0
creatinine (mg/dL)	0.8	0.2–0.7
urea (mg/dL)	31	17.1–45.0
Na (mmol/L)	137	132–145
K (mmol/L)	4.3	3.5–5.1
Ca (mEq/L)	4	4.58–5.32
AspAT (U/L)	24	15–40
ALT (U/L)	23	10–45
cholesterol (mg/dL)	118	106–224
triglycerides (mg/dL)	128	34–165
APTT (s)	38.42	28–40
INR	1.64	0.9–1.25
fibrinogen (g/L)	2.12	2.0–4.0
D-dimers	12,428.77	170–550
Urinalysis:	-	
protein (mg/dL)	120	-
RBC (HPF)	15–18	-
leukocytes (HPF)	0	-
24-h urine collection/protein (mg/kg/day)	24 (1.2 g/day)	-
IgA (mg/dL)		
IgG (mg/dL)	268.7	85–194
IgM (mg/dL)	869	706–1440
C3 (mg/dL)	60	44–113.1
C4 (mg/dL)	95	88–165
ASO	20.6	14–44
ANA	291	<200
ANCA	absent	-
	absent	-

## Data Availability

Not applicable.
